# Butyrolactone I Quantification from Lovastatin Producing *Aspergillus terreus* Using Tandem Mass Spectrometry—Evidence of Signalling Functions

**DOI:** 10.3390/microorganisms2020111

**Published:** 2014-06-04

**Authors:** Elina K. Palonen, Milla-Riina Neffling, Sheetal Raina, Annika Brandt, Tajalli Keshavarz, Jussi Meriluoto, Juhani Soini

**Affiliations:** 1 Biochemistry, Department of Biosciences, Åbo Akademi University, Artillerigatan 6, Åbo FI-20520, Finland; E-Mails: mneffling@gmail.com (M.-R.N.); annika.brandt@abo.fi (A.B.); jussi.meriluoto@abo.fi (J.M.); 2 Turku Centre for Biotechnology, University of Turku and Åbo Akademi University, Artillerigatan 6, Åbo FI-20520, Finland; E-Mail: juhani.soini@turkuamk.fi; 3 School of Life Sciences, University of Westminster, London W1W 6UW, UK; E-Mails: s.raina01@gmail.com (S.R.); T.Keshavarz@westminster.ac.uk (T.K.); 4 Faculty of Life Sciences and Business, Turku University of Applied Sciences, Lemminkäinengatan 30, Åbo FI-20520, Finland

**Keywords:** *Aspergillus terreus*, butyrolactone I, secondary metabolism, signalling, quorum sensing, HPLC, LC-ESI-MS-MS

## Abstract

*Aspergillus terreus* is an industrially important filamentous fungus producing a wide spectrum of secondary metabolites, including lovastatin and itaconic acid. It also produces butyrolactone I which has shown potential as an antitumour agent. Additionally, butyrolactone I has been implicated to have a regulating role in the secondary metabolism and morphology of *A. terreus*. In this study, a quantitative time-course liquid chromatography—electrospray ionisation—tandem mass spectrometry (LC-ESI-MS-MS) analysis of butyrolactone I is reported for the first time in nine-day long submerged cultures of *A. terreus*. Butyrolactone I was fragmented in the mass analysis producing a reproducible fragmentation pattern of four main daughter ions (*m*/*z* 307, 331, 363 and 393) in all the samples tested. Supplementing the cultures with 100 nM butyrolactone I caused a statistically significant increase (up to two-fold) in its production, regardless of the growth stage but was constitutive when butyrolactone I was added at high cell density during the stationary phase. Furthermore, the extracellular butyrolactone I concentration peaked at 48 h post inoculation, showing a similar profile as has been reported for bacterial quorum sensing molecules. Taken together, the results support the idea of butyrolactone I as a quorum sensing molecule in *A. terreus*.

## 1. Introduction

Many filamentous fungi, including *Aspergillus* spp., are prolific secondary metabolite producers with a wide variety of compounds ranging from pigments to mycotoxins with some having pharmaceutical or industrial benefits. *Aspergillus terreus* is a soil-dwelling saprophytic fungus producing a plenitude of secondary metabolites, including the toxins citrinin, emodin and gliotoxin, and other secondary metabolites such as aspulvinone, asterric acid, asterriquinones, butyrolactones, (+)-geodin, itaconic acid, mevinolin and sulochrin [1,2,3,4,5,6,7,8,9]. Some of the secondary metabolites produced by *A. terreus* have also pharmaceutical or industrial importance. For example, lovastatin, also known as mevinolin or monacolin K, is used as a serum cholesterol-lowering agent and is a competitive inhibitor of the 3-hydroxy-3-methylglutaryl-coenzyme A (HMG-Co A) reductase, which is the rate-limiting enzyme in the cholesterol biosynthesis [1]. *Aspergillus terreus* culturing is also utilized in the industrial production of itaconic acid, which is used in the production of polymers [10]. Furthermore, *A. terreus* is an emerging fungal pathogen being one of the fungal species causing invasive aspergillosis [11]. In order to improve the production of the pharmaceutically or industrially important secondary metabolites by various *A. terreus* strains, the growth conditions, culture media and biosynthetic clusters related to lovastatin and (+)-geodin have already been studied in great detail [12,13,14,15,16,17].

Kiriyama and co-workers [4] isolated and identified butyrolactone I (methyl 4-hydroxy-2-[[4-hydroxy-3-(3-methylbut-2-enyl)phenyl]methyl]-3-(4-hydroxyphenyl)-5-oxofuran-2-carboxylate; PubChem CID = 5235506; InChI = 1S/C24H24O7/c1-14(2)4-6-17-12-15(5-11-19(17)26)13-24(23(29)30-3)20(21(27)22(28)31-24)16-7-9-18(25)10-8-16/h4-5,7-12,2527H,6,13H2,1-3H3; InChIkey = NGOLMNWQNHWEKU-UHFFFAOYSA-N) from *A. terreus* cultures using several methods, including NMR, mass (MS) and ultraviolet (UV) spectra analyses. Since then, butyrolactone I has been found to inhibit CDK1 and CDK2 kinases, exhibiting antiproliferative activity against diverse tumour cell lines, e.g., lung cancer cells, pancreatic cancer cells and prostate carcinoma cells [18,19,20,21]. The biological role of butyrolactone I in the producing organism *Aspergillus terreus* has been studied by Schimmel *et al.* [12] and Raina *et al.* [17] whereby indications towards functioning as a quorum sensing-molecule were found. The addition of butyrolactone I to the *A. terreus* cultures was also found to cause an increase in secondary metabolite production, namely in lovastatin and sulochrin production [12]. Furthermore, Schimmel and co-workers [12] found that the addition of butyrolactone I in the *Aspergillus terreus* culture caused morphological differentiation similar to the changes caused by small γ-butyrolactone-containing molecules observed in filamentous bacteria belonging to the genus *Streptomyces*. Specifically, γ-butyrolactones such as A-factor and virginiae butanolides have been implicated as autoregulators in Streptomyces spp. where they have been shown to switch on secondary metabolism and morphological development [22]. Furthermore, Raina *et al.* [17] observed an increase in butyrolactone I production as a result of supplementing with butyrolactone I. Autoregulatory mechanisms and quorum sensing have been widely studied and are well established in bacteria whereas quorum sensing has only recently been discovered in some fungal species, including *Candida albicans*, *Aspergillus flavus* and *Aspergillus terreus* [17,23,24,25,26].

Quorum sensing signalling is mediated by small autoinducing molecules that are produced throughout the microbial growth, thus reflecting the cell density of the population. As the cell density reaches a threshold value, the accumulated autoinducer triggers the quorum sensing response, including the synthesis of the molecule itself, allowing the microbial population to act co-ordinately and adapt to the environmental conditions [26]. Further requirements for quorum sensing include dependency on population cell density and thereby on the growth stage of the organism [27]. Nevertheless, other factors such as timing and quorum sensing receptor availability may also contribute to the induction of quorum response [28]. Another important characteristic of a quorum sensing molecule is the restoration of the quorum response in an autoinducer-deficient mutant upon exogenous addition of the autoinducer [28,29]. Additionally, quorum sensing molecules regulate other physiological responses than metabolism of the molecule itself. Examples of such responses include development of bacterial biofilms, production of secondary metabolites and antibiotics, synthesis of virulence factors, sporulation and bioluminescence. Known autoregulatory or quorum sensing molecules include acyl-homoserine lactones, γ-butyrolactones and small peptides in bacteria and oxylipins and farnesol in fungi [22,23,24,25,30].

Butyrolactone I has been detected and quantified in *A. terreus* cultures using HPLC whereas no MS-MS data has been reported thus far [17,31]. Furthermore, no time-course quantitative MS-MS data for butyrolactone I has been reported. The production and detection of butyrolactone I is of interest due to its possible signalling role in *A. terreus* and its potential for utilization in the process of secondary metabolite production. In this study, the effect of butyrolactone I addition on its own production in *A. terreus*, strain MUCL38669 has been investigated to further elucidate its role in the growth process in lovastatin producing culture broth. The fragmentation pattern of butyrolactone I was obtained using LC-ESI-MS-MS and its production was followed in a time-course experiment by quantitating it with both HPLC and LC-ESI-MS-MS methods. Although time-course HPLC quantitation data of butyrolactone I are available [17], we report, in addition to the fragmentation pattern of butyrolactone I, a different view on this matter by analysing both HPLC and LC-ESI-MS-MS quantitation results. These results do not fully confirm the previously observed effects of exogenous butyrolactone I addition on the extracellular concentration of endogenous butyrolactone I. The quantitation data show an increase in butyrolactone I concentration as a result of the supplementation, which is in accordance with the idea that butyrolactone I may function as a quorum sensing molecule in *A. terreus*.

## 2. Experimental Section

### 2.1. Materials

The *Aspergillus terreus* strain MUCL38669 was obtained from CABI Biosciences UK Centre, Surrey, UK. Ingredients for the growth medium were purchased from Sigma-Aldrich Company Limited (Dorset, UK). The solvents in HPLC analysis (phosphoric acid, acetonitrile, water) and methanol used in the extraction of butyrolactone I were HPLC grade and from VWR International (Lutterworth, UK). The reagents for LC-ESI-MS-MS analysis (acetonitrile, formic acid, water) were all LC-MS grade and from Fluka, purchased from Sigma-Aldrich (Steinheim, Germany).

### 2.2. Culture Conditions

*A. terreus* strain MUCL38669 was batch cultured in three biological replicates for nine days. The medium composition was the same as used elsewhere in the production of secondary metabolites lovastatin, sulochrin and butyrolactone I [12,17]. *A. terreus* strain MUCL38669 spores were maintained on YME agar slants consisting of 4 g/L yeast extract, 10 g/L malt, 4 g/L glucose and 20 g/L agar. 10^7^ spores were used to inoculate 100 mL of inoculation medium (pH 6.8) consisting of 5 g/L corn steep liquor, 40 g/L tomato paste, 10 g/L oat flour, 10 g/L dextrose and 10 mL/L of trace element solution in a 250 mL Erlenmeyer flask. The trace element solution consisted of 1 g/L FeSO_4_·7H_2_O, 1 g/L MnSO_4_·4H_2_O, 0.025 g/L CuCl_2_·2H_2_O, 0.1 g/L CaCl_2_·H_2_O, 0.056 g/L H_3_BO_3_, 0.019 g/L (NH_4_)_6_Mo_7_O_24_·4H_2_O and 0.2 g/L ZnSO_4_·7H_2_O. The inoculation medium was incubated on a rotary shaker at 220 rpm for 25 h at 28 °C. Ten mL of the inoculation medium was used to inoculate 100 mL of GPY-L production medium consisting of 25 g/L glucose, 24 g/L peptonised milk, 2.5 g/L yeast extract and 50 g/L lactose in a 250 mL Erlenmeyer flask. The production medium was incubated on a rotary shaker at 220 rpm at 28 °C for up to 9 days. Butyrolactone I was added to the test sets 1, 2 and 3 at 24 h, 96 h and 120 h post inoculation, respectively. The butyrolactone I (BIOMOL International, Exeter, UK) to be added was dissolved in ethanol and was added to the test cultures to a final concentration of 100 nM (0.0425 µg/mL).

### 2.3. Extraction of Butyrolactone I from the Cultures

Duplicate 1 mL samples of the cultures were withdrawn at 24 h, 48 h, 96 h, 120 h, 144 h and 216 h post inoculation. The cells were separated from the growth medium by centrifuging the samples at 20 °C, 15,000 rcf for 20 min. One mL of methanol was added to both the cell pellets and culture supernatants and the samples were homogenised using a FastPrep-24 Instrument (MP Biomedicals, Cambridge, UK) for 30 min at 220 rpm to extract butyrolactone I. The homogenates were filtered through a 0.2 µm syringe filter prior to HPLC analysis or alternatively centrifuged at 10,000 rcf for 5 min prior to LC-ESI-MS-MS analysis to remove any particulate matter.

### 2.4. Butyrolactone I Assay

#### 2.4.1. HPLC

The equipment and detection parameters for butyrolactone I quantification from the methanol extracts by HPLC were as described elsewhere [17,32]. The quantification was done based on a concentration curve of external butyrolactone I standard (BIOMOL International, Exeter, UK). Dilution of the samples by the extraction procedure was taken into account by using approximate dilution factors when calculating the actual butyrolactone I concentration in the samples. The butyrolactone I concentration in the cell fraction was additionally normalized to the wet cell weight of the sample.

#### 2.4.2. LC-ESI-MS-MS

Butyrolactone I was detected and quantified from the duplicate methanol extracts by LC-ESI-MS-MS analysis. The instrumentation consisted of an Agilent Technologies (Waldbronn, Germany) 1200 Rapid Resolution (RR) LC coupled to a Bruker Daltonics High Capacity Trap Ultra Ion Trap MS with an electrospray (ESI) ion source (Bruker Daltonics, Bremen, Germany). The 1200 RR LC system included a vacuum degasser, model SL binary pump, refrigerated (set at 10 °C) autosampler, and column oven. The column was an Agilent Zorbax Eclipse XBD-C8 4.6 mm × 150 mm with 5 µm particle size. The mobile phase consisted of water (solvent A) and acetonitrile acidified with 0.1% formic acid (solvent B). The gradient used was from 10% acetonitrile to 90% over 10 min with the flow rate of 1 mL/min. The column was equilibrated back to 10% acetonitrile over 14 min with a flow rate of 1.5 mL/min. The total runtime was 27 min. The column was kept at 40 °C during the analysis. The injection volume was 1 µL. The ion source parameters were set as follows: dry temperature 365 °C, nebuliser pressure 60 psi and dry gas flow 11 L/min. The instrument ion optics was optimised for the *m*/*z* 425 with smart parameter settings. The ion trap was operated in the Ultra scan mode from *m*/*z* 220 to 500 with automatic MS-MS mode with mass range 422–428 *m*/*z* included. The fragmentation was achieved with fragmentation amplitude of 0.90 V with SmartFrag from 30% to 200% on. The ICC target was set to 300,000 with a maximum accumulation time of 100 ms.

### 2.5. Data Analysis

The LC-ESI-MS-MS data analysis was performed with Bruker Daltonics Data analysis software and Microsoft Excel. Butyrolactone I quantity was calculated based on daughter ion intensity areas for four daughter ions (*m*/*z* 307, 331, 363, 393). The signal response stability during the sample series run was monitored with external standards added into the sample list. In case needed, the signal area response was normalised according to the external standard signal response slope. For quantification with LC-ESI-MS-MS, a concentration curve of external butyrolactone I standard (Enzo Life Sciences, Lausanne, Switzerland) was used. Dilution of the samples by the extraction procedure was taken into account as above for HPLC analysis. The statistical significance of the observed differences was tested with Two Sample Student’s *t*-test using the statistical computing language and environment R version 2.11.1 [33].

## 3. Results and Discussion

### 3.1. Butyrolactone I Fragmentation Pattern

**Figure 1:**
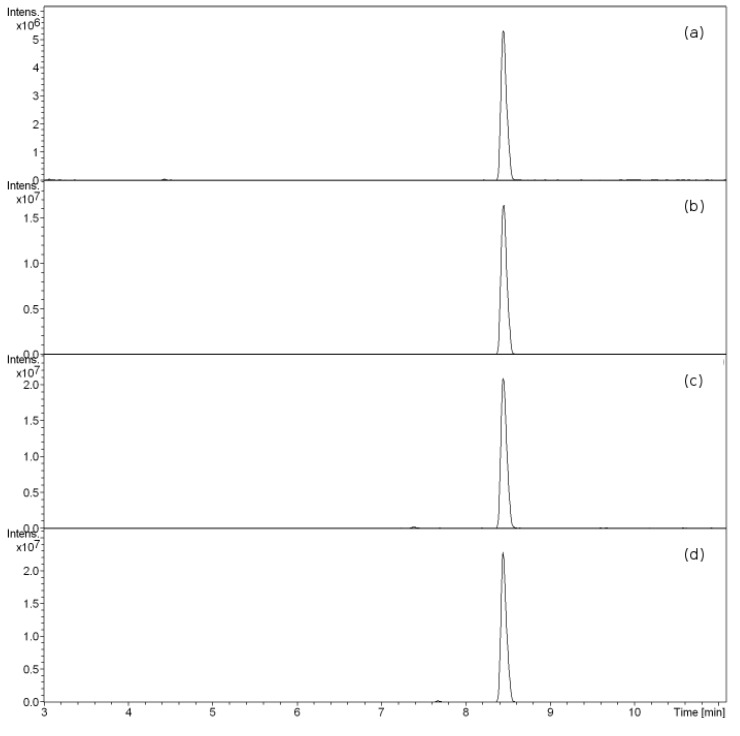
Detection and quantification of butyrolactone I from an intracellular fraction of unsupplemented *A. terreus* culture at 48 h post inoculation. Extracted ion chromatograms of the four main transitions of *m*/*z* 425.2: *m*/*z* (**a**) 307; (**b**) 331; (**c**) 363 and (**d**) 393 are shown and were used for quantification of butyrolactone I in this study.

**Figure 2:**
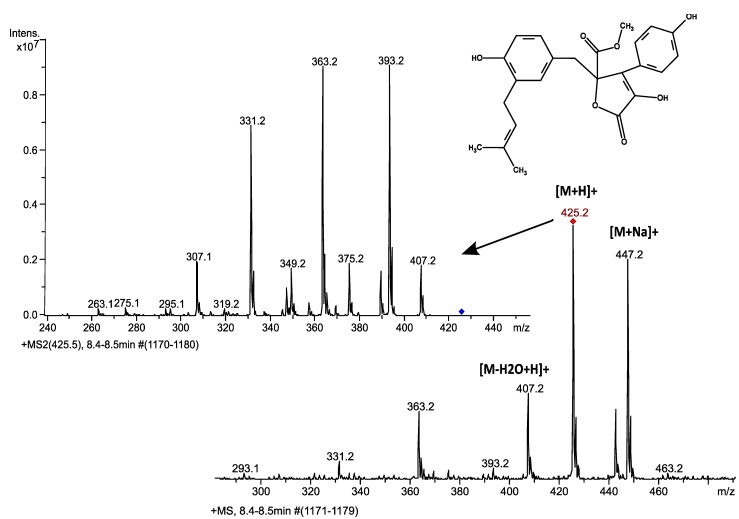
MS and MS-MS spectra for butyrolactone I detection on an LC-ESI-ion trap-MS instrument. This fragmentation pattern was seen clearly throughout the sample series. Four main fragments, *m*/*z* 307, 331, 363 and 393, were chosen for quantitative purposes.

*Aspergillus terreus* is known to produce butyrolactone I among its secondary metabolites [4]. Butyrolactone I was detected with LC-ESI-MS-MS both in the cells and culture supernatants from samples taken at six different time points during the nine-day long culturing. Butyrolactone I eluted with a retention time of 8.4 min in the gradient used, producing a clear and distinct peak with good signal to noise ratio with the selected transitions (Figure 1). The eluted butyrolactone I was detected in MS as the protonated ion, with sodium adduct as well as a fragment formed by a water loss (Figure 2). The protonated ion (*m*/*z* 425) was subsequently fragmented producing four main daughter ions (*m*/*z* 307, 331, 363 and 393) as shown in Figure 2. The produced daughter ions *m*/*z* 331 and *m*/*z* 393 can be calculated to result from a cleavage of either a hydroxyphenyl or an O-methyl group from butyrolactone I, giving molecular formulas [C_18_H_19_O_6_]^+^ and [C_23_H_21_O_6_]^+^, respectively. The fragment *m*/*z* 407, the result of water loss, was considered too unspecific to be included in the fragmentation pattern used for quantification purposes. The fragmentation pattern was consistently achieved in all the samples analysed verifying the production of butyrolactone I in the experimental conditions employed.

### 3.2. Butyrolactone I Quantification

**Figure 3:**
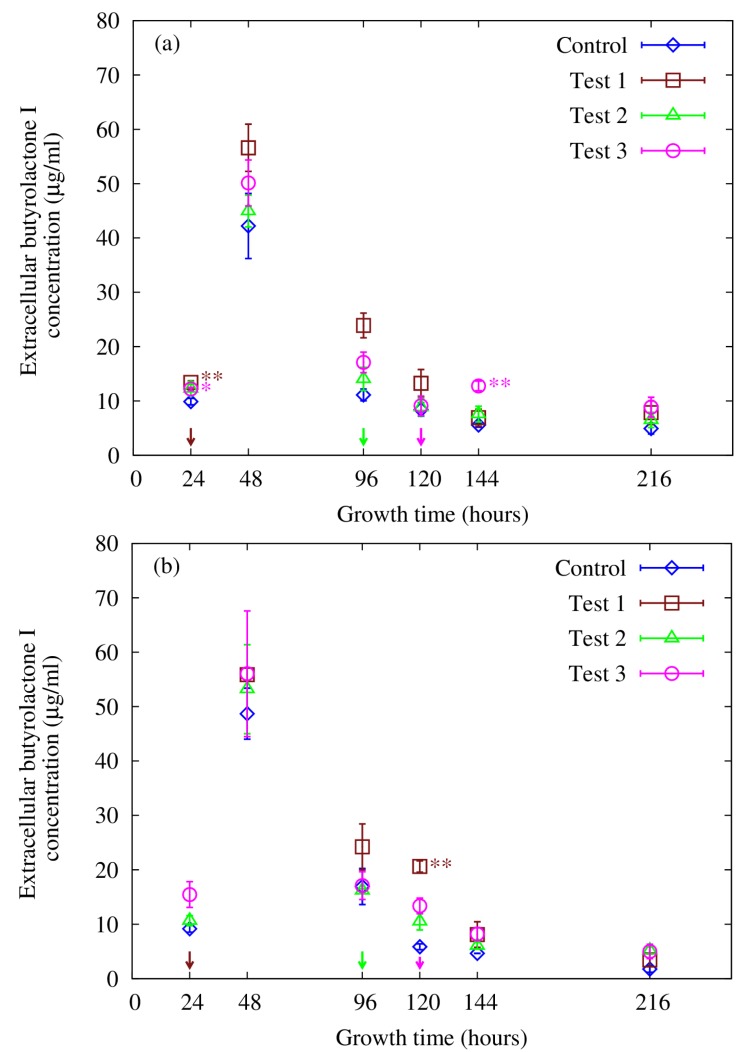
Butyrolactone I concentration in *A. terreus* culture supernatants as measured with (**a**) HPLC and (**b**) LC-ESI-MS-MS. Butyrolactone I was added to a final concentration of 100 nM (0.0425 µg/mL) to the *A. terreus* cultures at 24 h post inoculation (Test 1), 96 h post inoculation (Test 2) and 120 h post inoculation (Test 3). No butyrolactone I was added to the control set. The arrows indicate the time of butyrolactone I addition. The error bars represent standard error of the mean of three biological replicates. Statistically significant differences are denoted with the following statistical levels: * *p* < 0.05; ** *p* < 0.01.

**Figure 4:**
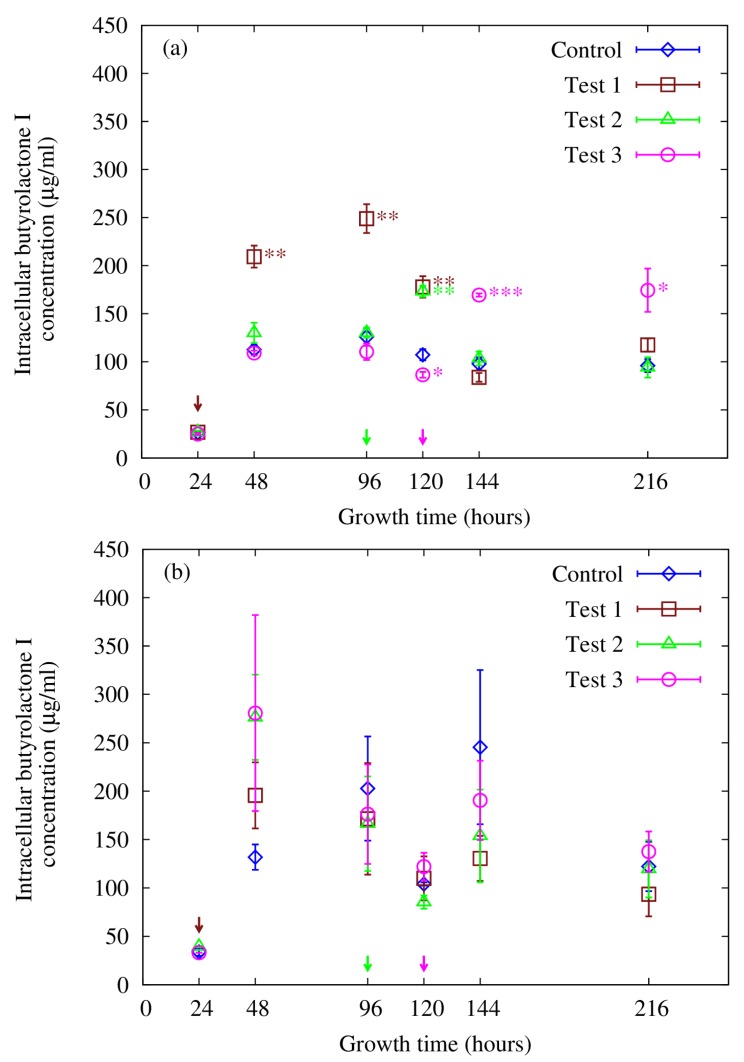
Butyrolactone I concentration in *A. terreus* cell pellets as measured with (**a**) HPLC and (**b**) LC-ESI-MS-MS. Butyrolactone I was added to a final concentration of 100 nM (0.0425 µg/mL) to the *A. terreus* cultures at 24 h post inoculation (Test 1), 96 h post inoculation (Test 2) and 120 h post inoculation (Test 3). No butyrolactone I was added to the control set. The arrows indicate the time of butyrolactone I addition. The error bars represent standard error of the mean of three biological replicates. Statistically significant differences are denoted with following statistical levels: * *p* < 0.05; ** *p* < 0.01; *** *p* < 0.001.

Butyrolactone I was separately quantified using both HPLC and LC-ESI-MS-MS analyses and eluted with a retention time of *ca.* 7.8 min in the conditions employed in the HPLC-UV analysis. Interestingly, *A. terreus* was found to have different butyrolactone I concentration profiles for extracellular and intracellular fractions (Figure 3 and Figure 4). Our setup for the LC-ESI-MS-MS analysis with an ion trap MS detection on the automatic MS-MS mode was well suited for the task: The fragmentation pattern obtained with a standard was seen in all the butyrolactone I-containing samples. Four transitions (*m*/*z* 307, 331, 363 and 393) gave clear peaks with little disturbance from the matrix, showing high specificity of the analysis, and were used in the quantification (see Figure 1). Signal fluctuation was not a concern during the runs, but was controlled for throughout the series with external standards. In spite of the consistent fragmentation pattern and clear elution peaks, the concentration profiles for the intracellular butyrolactone I showed higher variance amongst biological replicates. The intracellular butyrolactone I concentration values from the HPLC measurements turned out to have smaller variance—with relative standard error below 13%—than with LC-ESI-MS-MS measurements where the relative standard error exceeded 30%, which led to relying on the HPLC results regarding the intracellular butyrolactone I concentration.

### 3.3. Supplementing with Butyrolactone I—Effects on Extracellular Concentration

To further elucidate the role of butyrolactone I in *A. terreus*, a test series with four different batch culture sets supplemented with butyrolactone I to a final concentration of 100 nM (0.0425 µg/mL) was employed. The sets included a control set, where no butyrolactone I was added as well as three test sets where butyrolactone I was added at 24 h, 96 h and 120 h post inoculation. Time-course data on intracellular and extracellular butyrolactone I concentration in these sets was collected at six different time points during the culture. Small increases in the butyrolactone I concentration between the sets could be observed at 24 h post inoculation in the extracellular fractions before supplementation with butyrolactone I when compared to the control culture (Figure 3). Although statistically significant, these increases may not be biologically relevant as they occurred in the beginning of the culturing, resulting perhaps from minor differences in inoculation size. In addition, supplementing in the middle of the growth phase (at 24 h) and during the stationary phase (at 120 h) caused small and transient increases in extracellular butyrolactone I concentration—3.5-fold and 2.3-fold, respectively—when compared to control cultures 96 h and 24 h after supplementing (Test 1 in Figure 3b and Test 3 in Figure 3a). To our knowledge, these statistically significant (*p* < 0.01) increases have not been reported previously. Furthermore, the data in this current study do not show the highest increase in butyrolactone I production at 120 h post inoculation in the extracellular fractions that Raina *et al.* reported [17]. In that respect, the current data do not fully confirm the results reported by Raina *et al.* [17]. However, a distinct concentration maximum for butyrolactone I was observed in the culture supernatant at 48 h post inoculation in all the culturing sets, reaching a value of *ca.* 42 µg/mL and 49 µg/mL as measured with HPLC and LC-ESI-MS-MS, respectively (Figure 3). The concentration maximum at 48 h post inoculation in the culture supernatant was followed by an almost threefold decrease from 42 to 15 µg/mL and 49 to 17 µg/mL as measured with HPLC and LC-ESI-MS-MS, respectively.

### 3.4. Supplementing with Butyrolactone I—Effects on Intracellular Concentration

In contrast to the extracellular butyrolactone I concentration, a clear increase in butyrolactone I production could be seen in the intracellular concentration profiles as a result of supplementing the *A. terreus* culturing series with 100 nM (0.0425 µg/mL) butyrolactone I (Figure 4). The increase in intracellular butyrolactone I concentration was greatest when butyrolactone I was added in the middle of the growth phase at 24 h post inoculation, lasting up to 120 h post inoculation (*i.e.*, 96 h after supplementation), resulting in a statistically significant increase (*p* < 0.01) when compared to the unsupplemented control culture. The intracellular butyrolactone I concentration increased up to 2.0-fold reaching the highest value, *ca.* 250 µg/mL, 72 h after butyrolactone I supplementation at 24 h. Addition of butyrolactone I at the end of the fungal growth phase at 96 h post inoculation caused a statistically significant (*p* < 0.01) but transient and more subtle increase (1.6-fold) in butyrolactone I production. Supplementing at 120 h post inoculation, during the stationary phase when the cell density has reached its maximum value, caused an increase in the butyrolactone I production (up to 170 µg/mL) resulting in a statistically significant (*p* < 0.001) and 1.7-fold higher butyrolactone I concentration than in the control. Furthermore, butyrolactone I concentration in this set remained at a statistically significantly higher level (1.8-fold higher, *p* < 0.05) than in the control culture until the end of the culturing, *i.e.*, up to 216 h post inoculation. Supplementing in the mid-stationary phase caused a long-lasting increase (96 h in duration) in the butyrolactone I concentration, emphasizing the role of the growth stage at which butyrolactone I is added to the culture. The small decrease that can be observed in butyrolactone I concentration in the test set 3 at 120 h (*i.e.*, before butyrolactone I had been added) is most likely due to biological variation. In summary, it seems that when butyrolactone I is supplemented before the stationary phase the increase in butyrolactone I production is very transient and decreases before the stationary phase begins, whereas supplementation during the stationary phase causes a long-lasting increase in the production, implying that high cell density may be a prerequisite for the butyrolactone I production to remain at a higher level.

### 3.5. Intracellular Accumulation of Butyrolactone I, Autoinduction and Quorum Sensing

One of the fundamental characteristics of quorum sensing signalling molecules is the increase in concentration as the microbial population grows and the subsequent autoinduction when the population density threshold has been reached, which ensures the correct timing of the physiological response [26]. Schimmel and co-workers [12] have previously investigated the effect of butyrolactone I addition on the secondary metabolism of *A. terreus* and observed an increase in the production of both lovastatin and sulochrin in similar growth conditions and culture broth as was employed in this study. An almost threefold increase in lovastatin levels and an almost twofold increase in sulochrin levels were found when compared to the control where no butyrolactone I had been added. Furthermore, they observed morphological changes similar to the changes that were observed with small γ-butyrolactone-containing molecules, such as A-factor, in filamentous bacteria of the genus *Streptomyces*, leading Schimmel and co-workers [12] to suggest a role for butyrolactone I in the regulation of secondary metabolite production in *A. terreus*. The observed increase in lovastatin production upon butyrolactone I supplementation in their study is also consistent with the characteristics of quorum sensing molecules that induce the production of secondary metabolites in bacteria [22]. More recently, butyrolactone I was reported to improve lovastatin production, increase its own concentration and have a growth-stage specific response in *A. terreus* transcriptome as well [17]. However, to get better certainty and more reliable butyrolactone I identification in *A. terreus* cultures quantitative LC-ESI-MS-MS time-course data on butyrolactone I was collected during a nine-day long culturing. In order to test the hypothesis of its possible regulatory role, 100 nM butyrolactone I was added at different growth stages to the *A. terreus* cultures and subsequently quantified using both HPLC and LC-ESI-MS-MS methods. The separate analysis of the cell pellet and culture supernatant enabled the detection of butyrolactone I in both fractions, indicating secretion or free diffusion of butyrolactone I out of the cells. Remarkably, the overall extracellular and intracellular concentration profiles differed markedly from each other regardless of butyrolactone I addition.

Supplementation with butyrolactone I was found to cause a statistically significant increase in the intracellular fractions of all the culture sets when compared to the unsupplemented control set, strongly indicating an autoinducing role for butyrolactone I in *A. terreus*. The finding that the increase in butyrolactone I production was most long-lasting when butyrolactone I was added during the stationary phase (test 3 in Figure 4a) is in good agreement with the idea that the autoinduction creates a positive feedback loop as has been observed in quorum sensing bacteria [26]. The induction kinetics in this set implies that in the used experimental conditions the cell density reaches a high enough value during the stationary phase enabling the continuation of the increased production of butyrolactone I throughout the secondary metabolite production phase up to 216 h post inoculation, which has not been reported previously. Unfortunately, the analysis of the quantification data from the intracellular fractions obtained with LC-ESI-MS-MS was hampered by the high variance among the biological replicates leading to rely on the results obtained with HPLC. The more complex matrix (intracellular contents) may cause unspecific disturbance in HPLC-UV detection as well as signal suppression in LC-ESI-MS-MS detection. The higher variance in the LC-MS analysis is most probably due to the dynamic process of electrospray ionisation that is prone to matrix effects. Furthermore, estimating the possible effects of the complex matrix on the sample analysis was hampered by the lack of internal standard and by the use of only one injection per replicate sample. However, the measured values are in the same scale as obtained with HPLC, cross-validating both results in that respect. The same induction trend as seen with HPLC in this study has been reported although with lower concentration values [17]. Nevertheless, the MS gives higher certainty on compound identity and is a far more selective detection method than HPLC with UV detection, thus verifying the production of butyrolactone I in these experimental settings, which was one of the aims of this study. In summary, supplementing *A. terreus* fermentation with butyrolactone I resulted in a statistically significant increase in the production of butyrolactone I, indicative of autoinduction in accordance with other quorum sensing molecules found in bacteria [23,26].

### 3.6. Extracellular Butyrolactone I Is Depleted after 48 h Post Inoculation

To our knowledge, the high peak in the extracellular butyrolactone I concentration in the middle of the fungal growth has not been shown for any other secondary metabolite in *A. terreus*. Remarkably, a *Streptomyces griseus* signalling molecule, A-factor, which also contains a γ-butyrolactone moiety, displays a similar concentration profile having the concentration maximum at or near the middle of the exponential growth [34]. The starting material for A-factor biosynthesis is derived from intermediates in glycolysis and primary fatty acid biosynthesis and the A-factor concentration is suggested to reflect the biochemical and physiological state of the cells, leading to the activation of secondary metabolism and morphological differentiation after primary metabolism [22]. Phenylalanine or p-hydroxyphenylpyruvic acid have been suggested as starting material for butyrolactone I biosynthesis in *A. terreus* [35]. Analogously to the A-factor biosynthesis in *Streptomyces* spp., phenylalanine or p-hydroxyphenylpyruvic acid availability could reflect the physiological state of the cells as being derived or leaked from the aromatic acid biosynthesis pathways during primary metabolism. However, considering the intracellular accumulation of butyrolactone I over the culturing period, it does not seem likely that the extracellular depletion is due to a cessation in the biosynthesis.

In this study, the exogenously added butyrolactone I may inhibit secretion of endogenous butyrolactone I keeping the extracellular butyrolactone I concentration constant between all the culture sets. However, we observed a statistically significant increase in extracellular butyrolactone I concentration both at 120 h and 144 h post inoculation as a result of exogenous butyrolactone I. Possible inhibition or the mechanism of regulation was however not specifically studied here. As there was no possibility to use radioactively labelled precursors while the biosynthesis of butyrolactone I has not been very well documented, the added butyrolactone I cannot be distinguished from the endogenously produced molecule in the intra- and extracellular fractions. Nevertheless, the exogenously added amount of butyrolactone I is negligible in comparison with the measured increase both in the intra- and extracellular fractions indicating that the intracellular accumulation is of endogenous origin (supplementation of 100 nM = 0.0425 µg/mL *versus* the smallest intracellular increase of *ca.* 66 µg/mL is over 1000-fold and extracellular increase of *ca.* 7 µg/mL is over 100-fold). A nanomolar concentration of butyrolactone I was used similarly to what has been reported among others for A-factor, which is a γ-butyrolactone quorum sensing molecule in *S. griseus* [22]. Furthermore, if the supplemented butyrolactone I were actively pumped into the cells or were stuck to the cell walls, the resulting increase would be insignificant in comparison with the measured increase as the total amount of added butyrolactone I was 4.2 µg. To conclude, the butyrolactone I secretion is under tight regulation which is in accordance with the idea of its suggested role as a quorum sensing molecule.

### 3.7. Indications of Quorum Quenching?

Although butyrolactone I concentration steeply decreases after 48 h post inoculation, significant increases in extracellular butyrolactone I concentration could be observed during the stationary phase as a result of butyrolactone I supplementation (test 3 in Figure 3a and test 1 in Figure 3b). The increase in both sets is statistically significant but transient in contrast to what was observed in the intracellular fractions, perhaps due to signal suppression in the extracellular fractions. However, the decrease in butyrolactone I concentration in the extracellular culture medium after 48 h post inoculation can be seen in all the test sets regardless of butyrolactone I supplementation, suggesting that further butyrolactone I addition did not have any apparent influence on the molecule as studied in the extracellular fraction. This apparent contradiction with intracellular concentration may indicate growth stage-specific or -dependent differences in the signalling pathways between the intra- and extracellular parts. However, the purpose and mechanism of this extracellular depletion of butyrolactone I are still unclear and warrant further investigation. Quorum quenching enzymes or lactonases have been found to degrade autoinducer molecules and thus attenuate or regulate the quorum sensing signalling cascade [36]. This kind of quenching activity could possibly explain the mild effects on the extracellular butyrolactone I concentration in response to the supplementation with butyrolactone I. Butyrolactone I retained by the cells may have been protected from this degradation while being bound to its cognate quorum receptor molecule as has been suggested to be the case in *Agrobacterium tumefaciens* quorum sensing system [37]. Alternatively, the quorum quencher could have been secreted, exerting its function outside the cells, resulting in the degradation of extracellular quorum sensing molecules. This kind of extracellular quenching has been observed in one *Streptomyces* strain where an extracellular AHL-acylase was identified [38]. Moreover, the mechanism by which butyrolactone I is secreted or diffused out of the cells during the growth process is not known and remains to be further studied. Likewise, the means of regulating the extracellular and intracellular concentration of butyrolactone I is not known. To this end, transcriptome sequence data is currently being analysed in combination with microarray gene expression data of this *A. terreus* strain (MUCL 38669) in order to gain further insight into the role butyrolactone I has in this organism and to build a possible model for its mechanisms of action. Taken together, the results of this study imply that butyrolactone I is either secreted out of the cells only in the early stages of growth and is retained by the cells in the later stages of the growth, or degrades very rapidly once outside the cells. To our knowledge, this kind of change in the concentration of a fungal metabolite over the course of the fungal growth has not been reported previously and its mechanism and role in the culturing and secondary metabolism remains to be further characterized. The observed decrease in the extracellular butyrolactone I concentration after 48 h of growth may indicate extracellular quorum sensing signal turnover in order to restore the system and switch off the signalling.

## 4. Conclusions

The first LC-ESI-MS-MS detection and quantification of butyrolactone I from intra- and extracellular fractions of *A. terreus* cultures is reported, confirming its production in the experimental conditions employed. The LC-ESI-ion trap MS instrumentation was well suited for the task with several high abundance MS-MS fragments obtained with the automatic MS-MS function to provide good confidence in the analysis. HPLC-UV quantification, however, turned out to be more reliable in the analysis of intracellular butyrolactone I concentration, while the LC-ESI-MS-MS quantification results correlated well with the HPLC results in the extracellular fractions. Supplementing *A. terreus* cultures with butyrolactone I was found to significantly increase the production of butyrolactone I, indicating an autoinducing role for butyrolactone I in *A. terreus*. The extracellular butyrolactone I concentration displays a very distinct profile indicating extracellular signal suppression or quorum quenching further corroborating the suggested role of butyrolactone I in the regulation of secondary metabolism in *A. terreus*.
